# The role of IL-27 in susceptibility to post-influenza *Staphylococcus aureus* pneumonia

**DOI:** 10.1186/s12931-015-0168-8

**Published:** 2015-02-05

**Authors:** Keven M Robinson, Benjamin Lee, Erich V Scheller, Sivanarayana Mandalapu, Richard I Enelow, Jay K Kolls, John F Alcorn

**Affiliations:** Department of Pediatrics, Children’s Hospital of Pittsburgh of UPMC, Pittsburgh, PA USA; Department of Medicine, Dartmouth Medical School, Lebanon, NH USA; Richard K. Mellon Foundation Institute, Children’s Hospital of Pittsburgh of UPMC, Pittsburgh, PA USA; Department of Medicine, University of Pittsburgh Medical Center, Pittsburgh, PA USA

**Keywords:** IL-27, Influenza, *Staphylococcus aureus*, Type 17 immunity

## Abstract

**Background:**

Influenza is a common respiratory virus and *Staphylococcus aureus* frequently causes secondary pneumonia during influenza infection, leading to increased morbidity and mortality. Influenza has been found to attenuate subsequent Type 17 immunity, enhancing susceptibility to secondary bacterial infections. IL-27 is known to inhibit Type 17 immunity, suggesting a potential critical role for IL-27 in viral and bacterial co-infection.

**Methods:**

A murine model of influenza and *Staphylococcus aureus* infection was used to mimic human viral, bacterial co-infection. C57BL/6 wild-type, IL-27 receptor α knock-out, and IL-10 knock-out mice were infected with Influenza H1N1 (A/PR/8/34) or vehicle for 6 days followed by challenge with *Staphylococcus aureus* or vehicle for 24 hours. Lung inflammation, bacterial burden, gene expression, and cytokine production were determined.

**Results:**

IL-27 receptor α knock-out mice challenged with influenza A had increased morbidity compared to controls, but no change in viral burden. IL-27 receptor α knock-out mice infected with influenza displayed significantly decreased IL-10 production compared to wild-type. IL-27 receptor α knock-out mice co-infected with influenza and *S. aureus* had improved bacterial clearance compared to wild-type controls. Importantly, there were significantly increased Type 17 responses and decreased IL-10 production in IL-27 receptor α knock-out mice. Dual infected IL-10−/− mice had significantly less bacterial burden compared to dual infected WT mice.

**Conclusions:**

These data reveal that IL-27 regulates enhanced susceptibility to *S. aureus* pneumonia following influenza infection, potentially through the induction of IL-10 and suppression of IL-17.

## Background

Pneumonia is a significant cause of morbidity and mortality, and remains the leading cause of death among children worldwide. In the United States, influenza and pneumonia currently rank 8th overall as a cause of death, annually [1]. Numerous organisms can cause pneumonia, including viral pathogens, such as influenza, and gram-positive and gram-negative bacteria. One bacterial pathogen of particular concern is *Staphylococcus aureus,* which is a significant cause of both health care-associated and community-acquired pneumonia. The incidence of severe *S. aureus* pneumonia has been increasing, in part due to the emergence of community-acquired methicillin-resistant *S. aureus* (CA-MRSA) [2]. Preceding influenza infection is a well-recognized risk factor for bacterial pneumonia, including *S. aureus* pneumonia, and secondary bacterial pneumonia following influenza infection is a primary cause of influenza-related death [3-5]. However, full understanding of the complex immunological mechanisms leading to this enhanced susceptibility remains elusive. Influenza-associated impairment of inflammatory pathways required for protective anti-bacterial host defense is the subject of ongoing investigation.

Interleukin-27 (IL-27) is a heterodimeric cytokine with the ability to inhibit multiple T-cell pathways, including Type 17 responses [6]. Type 17 immunity is associated with a neutrophilic inflammatory response via its signature effector cytokines IL-17 and IL-22 in response to stimulation by multiple signals, including IL-23, IL-1β, and IL-6 [7]. Previous data suggest that IL-27 impairs the Type 17 response by inhibiting T_H_17 differentiation via STAT-1 dependent blockade of retinoid-related orphan receptor (ROR)-γT up-regulation in naïve T-cells [8]. In addition, IL-27 is a major stimulus for T-cell production of IL-10, a broadly expressed anti-inflammatory cytokine with a range of downstream effects [6,9,10]. Recently, IL-10 was shown to decrease lung pathology in a murine model of severe influenza infection [11]. However, IL-10 has also been implicated as a mediator of enhanced lung susceptibility to numerous bacterial infections, including secondary *Streptococcus pneumoniae* infection following influenza, primary *Klebsiella pneumoniae* pneumonia, and *Pseudomonas aeruginosa* sepsis in murine models [12-14]. Together, these data suggest that IL-10 mediated suppression of lung pathology following viral infection may come at the cost of enhanced susceptibility to secondary bacterial super-infection.

Previously, we have shown that influenza-mediated inhibition of Type 17 immunity enhanced susceptibility to secondary *S. aureus* pneumonia in mice [15,16]. In these studies we demonstrated that preceding influenza infection resulted in decreased IL-1β and IL-23 expression and decreased subsequent IL-17 and IL-22 production. Exogenous IL-1β or IL-23 resulted in improved IL-17 and IL-22 expression in co-infected mice and improved *S. aureus* clearance. Little is currently known about the role of IL-27 in pneumonia. Since IL-27 has been shown to inhibit Type 17 responses, we further explored the contribution of IL-27 in a mouse model of influenza infection, *S. aureus* pneumonia, and influenza and *S. aureus* co-infection. To test this, we utilized IL-27 receptor α knock-out (IL-27Rα−/−) mice and examined the impact of the IL-27 signaling on viral, bacterial and co-infection pathogenesis.

## Methods

### Mice

Six to eight week old male IL-10−/− mice and age matched wild-type, C57BL/6 mice were purchased from The Jackson Laboratory (Bar Harbor, Maine). Additional wild-type, C57BL/6 mice were purchased from Taconic Farms (Germantown, NY). IL-27Rα−/− mice were generated as previously described [17] and generously provided by Nico Ghilardi (Genentech, South San Francisco, CA). Mice were maintained under pathogen-free conditions within the animal facilities at the Children’s Hospital of Pittsburgh of UPMC. All of the studies were performed on age- and sex-matched mice. Animal studies were conducted with approval from the University of Pittsburgh Institutional Animal Care and Use Committee.

### *S. aureus* infection

Methicillin-sensitive *S. aureus* (American Type Culture Collection (ATCC 49775) producing γ-hemolysin and Panton-Valentine leukocidin was purchased from the ATCC. Methicillin-resistant *S. aureus* (USA300) was obtained as gift from Dr. Alice Prince (Columbia University, New York, NY). *S. aureus* was cultured as detailed by ATCC recommendations in casein hydrolysate yeast extract containing-modified medium overnight for 18 hours to stationary growth phase. Mice were inoculated with 1×10^8^ CFU, or 5×10^7^ CFU for USA300, (in 50 μl sterile PBS) by oropharyngeal aspiration, and lungs were harvested 24 hours later.

### Influenza A/PR/8/34 H1N1 infection

Influenza A/PR/8/34 H1N1 was propagated in chicken eggs as previously described [18]. Mice were infected with 100 PFU of influenza A/PR/8/34 H1N1 (in 40 μl sterile PBS) from a frozen stock or control PBS by oropharyngeal aspiration. Infected mice were incubated for 6 days, at which time mice received *S. aureus* inoculum or control PBS. After an additional 24 hours, lungs were harvested. Viral burden was determined by quantitative real-time RT-PCR on lung RNA for viral matrix protein as described previously [15].

### Analysis of lung inflammation

At the indicated time points, mouse lungs were lavaged with sterile PBS for inflammatory cell differential counts. The cranial lobe of the right lung was homogenized in sterile PBS by mechanical grinding. The resulting lung homogenate was used for bacterial colony counting and cytokine analysis by Lincoplex (Millipore, Billerica, MA) and Bioplex (Bio-Rad Laboratories, Richmond, CA). The middle and caudal lobes of the right lung were homogenized under liquid nitrogen for RNA isolation by TRIzol RNA extraction. RNA analysis was performed by quantitative RT-PCR using Assay on Demand Taqman probes and primers (Applied Biosystems, Foster City, CA). PCR assays were normalized to the house keeping gene hprt. The left lung was fixed in 10% neutral buffered formalin for histopathology scoring of H&E stained sections. Scoring was conducted by two blinded scorers using a 0–4 scale (4 being the most inflammation, 0 the least) for parenchymal, peribronchial, and perivascular inflammation.

### Statistical analysis

All of the data are presented as the mean +/− SEM. Significance was tested by unpaired t test (for two means), or two-way ANOVA (for multiple data groups) followed by Bonferroni’s post-test. Data was analyzed using the GraphPad Prism and/or Microsoft Excel software packages.

## Results

### IL-27 regulates morbidity and cytokine production during influenza infection

**Figure 1:**
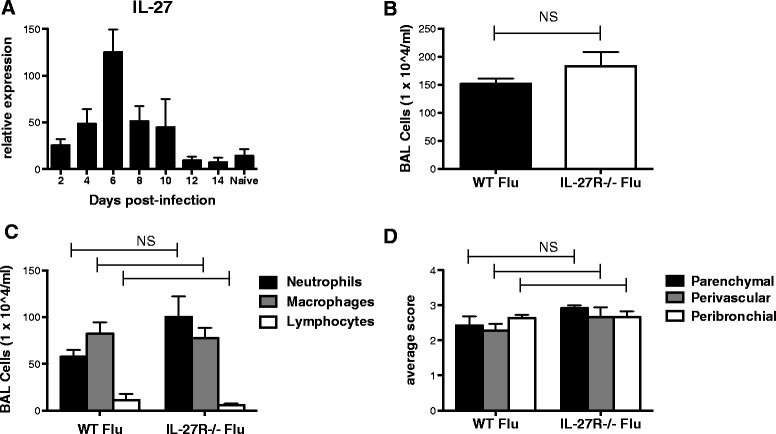
**IL-27 regulates morbidity during influenza infection.** C57BL/6 mice were infected with 100 PFU of influenza A PR/8/34 and sacrificed at days 2–14 post-infection. IL-27 mRNA gene expression in lung tissue was measured by RT-PCR, n = 3-4 **(A)**. C57BL/6, WT and IL-27Rα−/− mice, were infected with 100 PFU of influenza A PR/8/34 for 7 days. Cellular inflammation was examined by total cells per milliliter in the bronchoalveolar lavage fluid (BAL) **(B)**, cell differentials **(C)** and hematoxylin and eosin-stained lung sections that were quantified for lung inflammation (KMR and JFA) blinded to the groups **(D)**, n = 7. *p < 0.05 IL-27Rα−/− versus WT mice. All data are reported as means ± SEM for each group by unpaired t test **(B)** and two-way analysis of variance with the Bonferroni’s post-test **(C, D)**. Data are the combined results of two replicate experiments.

**Figure 2:**
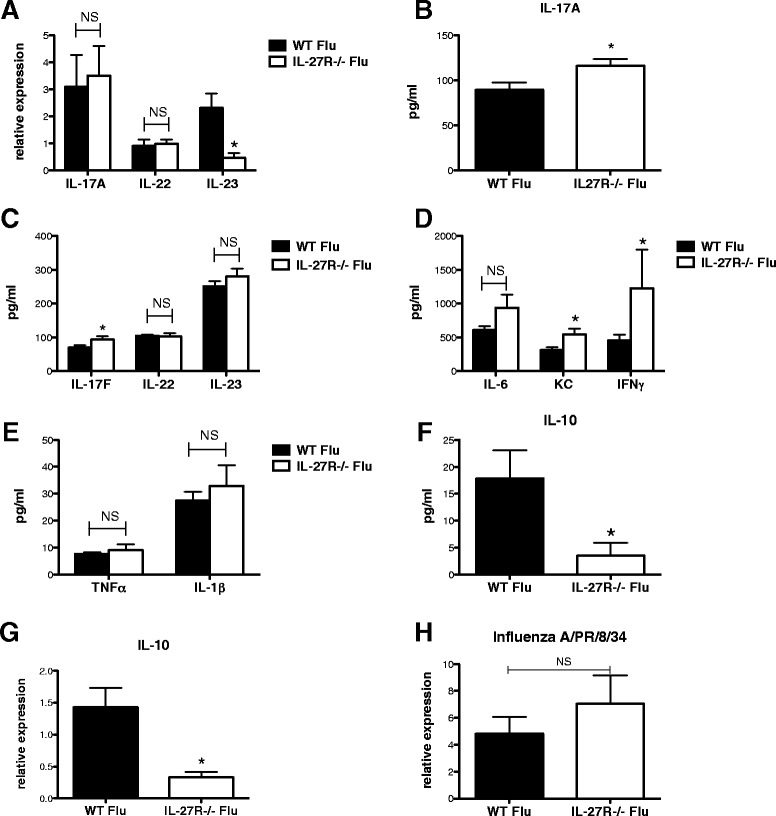
**IL-27 regulates cytokine production during influenza infection.** C57BL/6, WT and IL-27Rα−/− mice, were infected with 100 PFU of influenza A PR/8/34 for 7 days. Type 17-associated inflammatory cytokine mRNA gene expression in lung homogenate was measured by RT-PCR, n = 7 **(A)** and cytokine/chemokine levels were measured in lung homogenate by Lincoplex, n = 7 **(B-F)**. IL-10 mRNA gene expression in lung homogenate was measured by RT-PCR, n = 7 **(G)**. Viral burden was determined by measuring influenza matrix protein mRNA gene expression in lung homogenate by RT-PCR, n = 7 **(H)**. *p < 0.05 IL-27Rα−/− versus WT mice. All data are reported as means ± SEM for each group by unpaired t test. Data are the combined results of two replicate experiments.

First, we examined the role of IL-27R signaling in the host response to influenza infection. C57BL/6 wild-type (WT) mice were infected with 100 PFU of influenza A PR/8/34 H1N1 and sacrificed at days 2–14 post-infection. IL-27 mRNA levels increased following infection with influenza, peaking on day 6 post-infection and returning to baseline by day 12 (Figure 1A). Next, C57BL/6, WT and IL-27Rα−/− mice, were challenged with influenza A/PR/8/34 H1N1 for 7 days. Weight loss was used as a parameter to measure morbidity in response to viral infection. IL-27Rα−/− mice lost significantly more weight than WT mice by day 6 post-infection (data not shown). There were no significant differences in inflammatory cell recruitment to the airways or lung inflammation scores between the two groups (Figure 1B-D). Thereafter, we examined inflammatory cytokine production in the lung. There was no significant difference in IL-17 or IL-22 mRNA expression, however there was decreased IL-23 mRNA expression in the IL-27Rα−/− mice compared to WT mice (Figure 2A). On examination of Type 17-associated cytokine protein levels, there was an increase in both IL-17A and IL-17 F in the IL-27Rα−/− mice compared to WT mice (Figure 2B-C). Despite the decrease in IL-23 mRNA expression, there was no significant difference in IL-23 protein levels between the two groups. This is likely the result of timing as mRNA and protein levels were both assessed at a single time point following bacterial challenge. Next, neutrophil chemokine and cytokine activators keratinocyte chemoattractant (KC) and interferon IFNγ were examined and found to be increased in the IL-27Rα−/− mice compared to WT mice (Figure 2D). However, there was no significant difference in TNFα, IL-6 and IL-1β production between the two groups (Figure 2D-E). Interestingly, IL-10 (both mRNA and protein production) was significantly attenuated in the IL-27Rα−/− mice compared to WT mice (Figure 2 F-G). We found no differences in viral load between WT mice and IL-27Rα−/− mice infected with influenza (Figure 2H). Our findings demonstrate that IL-27 may partially regulate IL-17 and IL-10 production during influenza infection. These data demonstrate the role of IL-27 signaling in host defense against influenza.

### IL-27 contributes to cellular inflammation but not clearance of S. aureus pneumonia

**Figure 3:**
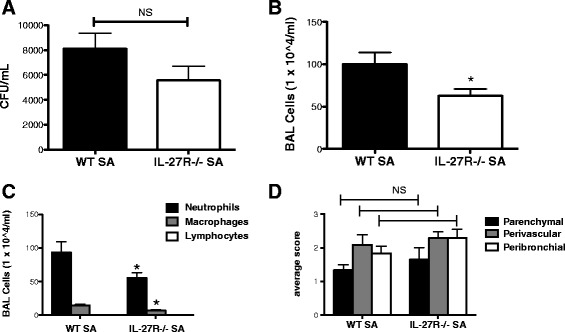
**IL-27 signaling alters cellular inflammation but not clearance of bacteria during** ***S. aureus*** **pneumonia.** C57BL/6, WT and IL-27Rα−/− mice, were infected with 1x10^8^ of *S. aureus* (ATTC 49775) for 24 hours. Bacterial colony counts in the lung were measured, n = 7 **(A)**. Cellular inflammation was examined by total cells per milliliter in the BAL **(B)**, cell differentials **(C)** and hematoxylin and eosin-stained lung sections that were quantified for lung inflammation by a pathologist (KMR and JFA) blinded to the groups **(D)**, n = 7. *p < 0.05 IL-27Ra−/− versus WT mice. All data are reported as means ± SEM for each group by unpaired t test **(A-C)** and two-way analysis of variance with the Bonferroni’s post-test **(D)**. Data are the combined results of two replicate experiments.

**Figure 4:**
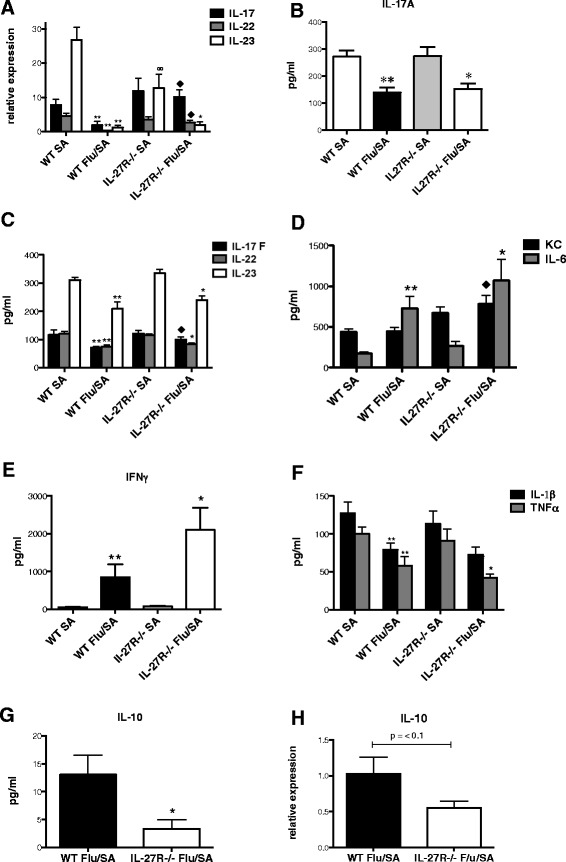
**IL-27 signaling affects IL-10 production during influenza,** ***S. aureus*** **co-infection.** C57BL/6, WT and IL-27Rα−/− mice received 100 PFU of influenza A PR/8/34 or vehicle (PBS) for 6 days followed by 1x10^8^ of *S. aureus* (ATTC 49775) or vehicle (PBS) for 24 hours. Type 17-associated inflammatory cytokine mRNA gene expression was measured in lung homogenate by RT-PCR, n = 7 **(A)** and cytokine/chemokine levels in lung homogenate were measured by Lincoplex, n = 7 **(B-G)**. IL-10 mRNA expression in lung homogenate as measured by RT-PCR **(H)**. Data are the combined results of two replicate experiments. *p < 0.05 IL-27Rα−/− mice infected with *S. aureus* versus influenza, *S. aureus*. **p < 0.05 C57BL/6 mice infected with *S. aureus* versus influenza, *S. aureus*. ∞p < 0.05 IL-27Rα−/− mice infected with *S. aureus* versus C57BL/6 mice infected with *S. aureus*. ♦p < 0.05 IL-27Rα−/− mice co-infected with influenza, *S. aureus* versus C57BL/6 mice co-infected with influenza, *S. aureus*. All data are reported as means ± SEM for each group by unpaired t test.

Next, we examined the impact of IL-27 signaling on *S. aureus* infection in the lung. To do this, we first examined IL-27 mRNA expression in WT mice challenged with *S. aureus* (ATTC 49775) for twenty-four hours compared to vehicle challenged mice. We found that *S. aureus* induced IL-27 mRNA 24 hours following challenge (approximately 7.5 fold, data not shown). Next, WT and IL-27Rα−/− mice were challenged with *S. aureus* (ATTC 49775) for 24 hours. Analysis was performed at 24 hours based on our previous experience in which bacterial burden, lung injury, and immune activation consisting of both innate cells and T cells can all be effectively assessed at 24 hours [19]. There was no significant difference in bacterial clearance between the WT and IL-27Rα−/− mice that received *S. aureus* alone (Figure 3A), suggesting that IL-27 signaling does not play a major role in *S. aureus* host defense. Interestingly, there was decreased total cell, neutrophil and macrophage recruitment to the airways in IL-27Rα−/− mice compared to WT mice (Figure 3B-C). Histopathology showed no significant difference in lung inflammation between the two groups (Figure 3D). Consistent with a limited impact on inflammation, IL-27Rα−/− mice displayed no changes in cytokine or chemokine production compared to WT with the exception of IL-23 mRNA expression (Figure 4A-F). Although there was significantly decreased IL-23 mRNA expression (Figure 4A) in the IL-27Rα−/− mice, there were no changes observed in protein levels (Figure 4C). Our findings demonstrate that IL-27 signaling has a limited role, if any, in *S. aureus* infection in the murine lung.

### IL-27 signaling influences bacterial clearance and cellular inflammation in influenza, S. aureus co-infection

**Figure 5:**
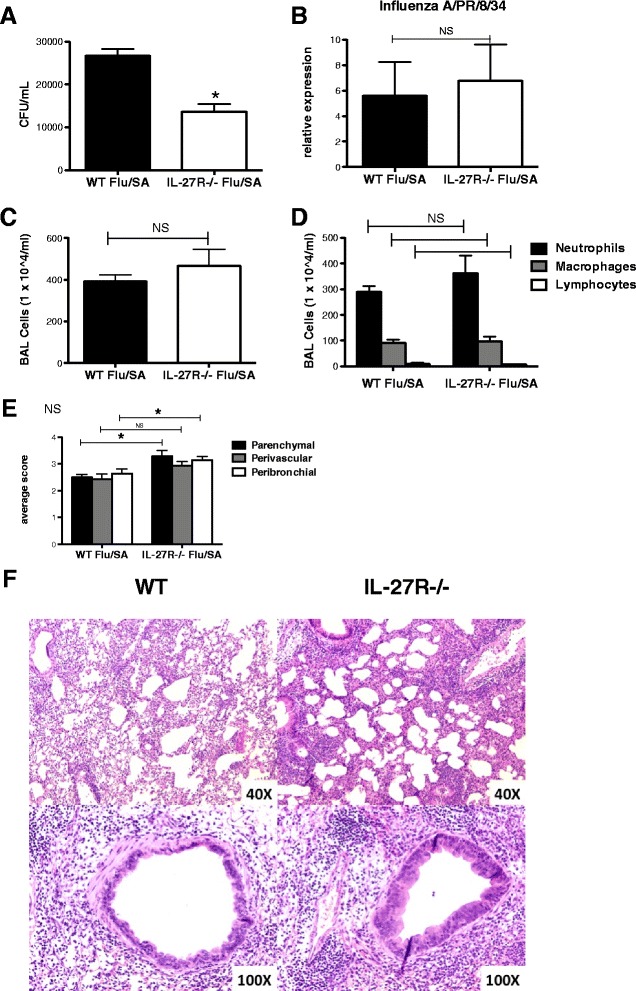
**IL-27 signaling influences bacterial clearance and cellular inflammation in influenza,** ***S. aureus*** **co-infection.** C57BL/6, WT and IL-27Rα−/− mice, received 100 PFU of influenza A PR/8/34 or vehicle (PBS) for 6 days followed by 1x10^8^ of *S. aureus* (ATTC 49775) or vehicle (PBS) for 24 hours. Bacterial colony counts in the lung were measured, n = 7 **(A)**. Viral burden was determined by the measurement of influenza matrix protein mRNA gene expression in lung homogenate by RT-PCR, n = 7 **(B)**. Cellular inflammation was examined by total cells per milliliter in the BAL **(C)**, cell differentials **(D)** and hematoxylin and eosin-stained lung sections that were quantified for lung inflammation (KMR and JFA) blinded to the groups **(E)** with representative sections shown **(F)**, n = 7. *p < 0.05 IL-27Rα−/− versus WT mice. All data are reported as means ± SEM for each group by unpaired t test **(A-D)** and two-way analysis of variance with the Bonferroni’s post-test **(E)**. Data are the combined results of two replicate experiments.

Preceding influenza is a significant risk factor for the development of secondary *S. aureus* pneumonia. Influenza and bacterial co-infection is a principal cause of influenza-related death. Since IL-27 signaling modulated influenza host defense, we investigated the role of IL-27 in viral, bacterial co-infection by challenging C57BL/6 and IL-27Rα−/− mice with influenza A/PR/8/34 H1N1 or vehicle (PBS) for 6 days followed by *S. aureus* (ATTC 49775) or vehicle (PBS). Twenty-four hours following bacterial challenge, burden was assessed. As expected, there was significantly decreased clearance of *S. aureus* in the WT mice that were infected with preceding influenza, compared to the WT mice that received *S. aureus* alone (Figures 3A, 5A). Bacterial burden was increased in co-infected mice by approximately 3-fold, similar to our previous findings [15]. In the IL-27Rα−/− mice, there was also significantly decreased clearance of *S. aureus* in the mice that received preceding influenza compared to the mice that received *S. aureus* alone. In the IL-27Rα−/− mice bacterial burden was increased approximately 2.5-fold (Figures 3A, 5A). Although preceding influenza enhanced susceptibility to secondary *S. aureus* pneumonia in both WT and IL-27Rα−/− mice, the bacterial burden was significantly reduced in the co-infected IL-27Rα−/− mice compared to co-infected WT mice, suggesting that IL-27 signaling plays a role in the development of secondary *S. aureus* pneumonia in influenza, *S. aureus* co-infection. Viral load of influenza was not significantly different between the two groups (Figure 5B). Our findings demonstrate that lack of IL-27 signaling results in improved clearance of *S. aureus* during influenza, *S. aureus* co-infection. However, IL-27 does not seem to play a direct role in exacerbation of *S. aureus* by influenza virus, as WT and IL-27Rα−/− mice had similar fold exacerbation of *S. aureus* secondary infection.

To further investigate the role of IL-27 in bacterial host defense, we examined lung inflammation in our influenza, *S. aureus* co-infection model. There was no significant difference in inflammatory cell recruitment to the airways between the co-infected WT and IL-27Rα−/− mice (Figure 5C-D). However, both WT and IL-27Rα−/− co-infected mice had significantly increased airway inflammation compared to mice singularly infected with *S. aureus* or influenza (Figures 1D, 3C, 5D). Histology revealed increased parenchymal, peribronchial, and perivascular inflammation in both WT and IL-27Rα−/− co-infected mice compared to mice singularly infected with *S. aureus* (Figures 3D, 5E-F). In addition, IL-27Rα−/− co-infected mice had increased parenchymal and peribronchial inflammation compare to co-infected WT mice (Figure 5E-F). We observed increased gene expression of IL-17 and IL-22 in the co-infected IL-27Rα−/− mice compared to co-infected WT mice (Figure 4A). However, there was no change in IL-23 between the two groups. In addition, there was attenuation of gene expression of the Type 17 effector cytokines, IL-17, IL-22, and IL-23 in the influenza, *S. aureus* infected WT mice compared to WT mice that received *S. aureus* alone, similar to previously published data [15]. There was no significant difference in IL-17 and IL-22 gene expression in the co-infected IL-27Rα−/− mice compared to IL-27Rα−/− mice that received *S. aureus* alone, however, there was decreased expression of IL-23 in the co-infected IL-27Rα−/− mice compared to IL-27Rα−/− mice that were singularly infected with *S. aureus*. IL-17A and IL-17 F protein levels were decreased in the co-infected IL-27Rα−/− mice compared to IL-27Rα−/− mice that were singularly infected with *S. aureus* (Figure 4B-C)*.* Similarly, IL-22 and IL-23 protein levels were decreased in the co-infected IL-27Rα−/− mice compared to IL-27Rα−/− mice that were singularly infected with *S. aureus* (Figure 4C). Replicating our previously published data [15] there was attenuation of production of the Type 17 effector cytokines, IL-17A, IL-17 F, IL-22, and IL-23 in the influenza, *S. aureus* infected WT mice compared to WT mice that received *S. aureus* alone (Figure 4C).

Following examination of the Type 17 cytokines, we assessed levels of neutrophil chemokine and cytokine activators. Production of the Type 17 cytokine-associated chemokine KC was increased in the co-infected IL-27Rα−/− mice compared to co-infected WT mice. However, there was no significant difference between co-infected WT mice compared to *S. aureus* infected WT mice or co-infected IL-27Rα−/− mice compared to *S. aureus* infected IL-27Rα−/− mice (Figure 4D). Although there was no significant difference in IFNγ production between the co-infected IL-27Rα−/− mice compared to co-infected WT mice, there was increased IFNγ production in the co-infected WT mice compared to *S. aureus*-infected WT mice and in the co-infected IL-27Rα−/− mice compared to *S. aureus*-infected IL-27Rα−/− mice (Figure 4E). Next, we examined production of IL-6 and TNFα, both of which are indicators of general inflammation There was no significant difference in IL-6 between the co-infected IL-27Rα−/− and WT mice, although there was increased IL-6 in the co-infected WT mice compared to *S. aureus*-infected WT mice and increased IL-6 in the co-infected IL-27Rα−/− mice compared *S. aureus*-infected IL-27Rα−/− mice (Figure 4D). There was no significant difference in TNFα between the co-infected IL-27Rα−/− and WT mice, although there was decreased TNFα in the co-infected WT mice compared to *S. aureus*-infected WT mice and decreased TNFα in the co-infected IL-27Rα−/− mice compared *S. aureus*-infected IL-27Rα−/− mice (Figure 4F). There was no consistent pattern observed between the trends of IL-6 and TNFα, suggesting no difference in general inflammation between the WT and IL-27Rα−/− mice. In addition, IL-6 is known to activate both STAT 3 and IL-23R expression [20,21], which could potentially play a role in IL-17 expression during influenza and *S. aureus* infection. Next, we examined IL-1β production, which is known to influence polarization of naïve CD4^+^ cells into T_H_17 cells. We observed no difference in IL-1β expression between groups, except for co-infected WT mice compared to *S. aureus*-infected WT mice, which we have previously shown (Figure 4F) [15].

**Figure 6:**
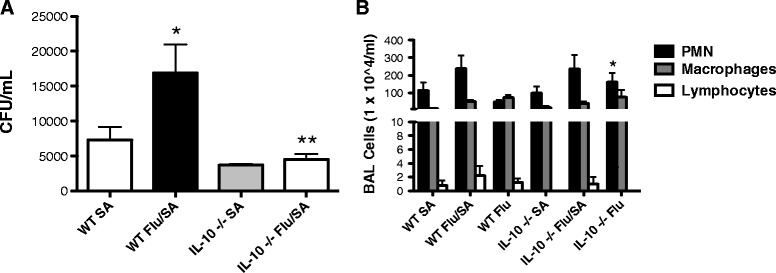
**IL-10 influences bacterial burden during influenza,** ***S. aureus*** **co-infection.** IL-10−/− mice received 100 PFU of influenza A PR/8/34, followed by challenge with *S. aureus* (USA 300) at 6 days post-influenza. Bacterial burden was assessed 24 hours following bacterial challenge **(A)**, n = 4. Cellular inflammation was examined by differentials of total cells per milliliter in the BAL fluid **(B)**, n = 4. *p < 0.05 C57BL/6 mice infected with *S. aureus* versus influenza, *S. aureus*. **IL-10−/− mice infected with influenza, *S. aureus* compared to WT mice infected with influenza, *S. aureus*. All data are reported as means ± SEM for each group by unpaired t test.

Although prior studies have shown mixed results, there is evidence in a post-influenza pneumococcal pneumonia model that IL-10 contributes to enhanced susceptibility to secondary bacterial pneumonia [12]. Because IL-10 production was severely decreased in influenza infected IL-27Rα−/− mice compared to controls, we proposed that IL-27 induction of IL-10 might play a role in the development of secondary *S. aureus* pneumonia. We examined levels of IL-10 production in both WT and IL-27Rα−/− mice co-infected with influenza, *S. aureus*. We observed significantly decreased levels of IL-10 gene expression (p < 0.1) and production (p < 0.05) in the co-infected IL-27Rα−/− mice compared to WT mice (Figure 4G-H). Mice singularly infected with *S. aureus* did not produce measurable levels of IL-10. We hypothesized that decreased IL-10 production in IL-27Rα−/− mice may contribute to enhanced inflammation, but improved *S. aureus* clearance. To test this, IL-10−/− mice received influenza A/PR/8/34 H1N1 or vehicle (PBS), followed by *S. aureus* (USA 300) or vehicle (PBS) at 6 days post-influenza. Twenty-four hours following bacterial challenge, bacterial burden was assessed. Although there was increased bacterial burden in the co-infected WT mice compared to WT mice that received *S. aureus* alone, there was no difference in the co-infected IL-10−/− mice compared to the IL-10−/− mice that received *S. aureus* alone (Figure 6A). Furthermore, the dual infected IL-10−/− mice cleared bacteria much more effectively compared to dual infected WT mice. In the singularly influenza-infected IL-10−/− mice, there was increased neutrophilia compared to the influenza-infected WT mice (6B). However, no difference in inflammatory cell recruitment to the airways was seen between the co-infected IL-10−/− mice compared to WT mice.

## Discussion

This study suggests a potential role for IL-27 signaling during viral and bacterial co-infection. First, we show that IL-27 mRNA levels increase during influenza infection, peaking at day 6 post-infection and returning to baseline by day 12 post-infection. IL-27Rα−/− mice have increased weight loss and increased production of IL-17A and IL-17 F compared to control mice. In addition, in the absence of IL-27 signaling, IL-10 production during influenza infection is attenuated. Next, we illustrate decreased neutrophil and macrophage recruitment to the airways during *S. aureus* infection in IL-27Rα−/− mice. However, there was little impact of IL-27 signaling on host defense against *S. aureus* in the absence of preceding influenza. Finally, we investigated influenza, *S. aureus* co-infection and IL-27Rα−/− mice exhibited increased bacterial clearance compared to controls. Despite improved bacterial clearance, IL-27Rα−/− mice displayed increased inflammation on histopathology. Co-infected IL-27Rα−/− mice also exhibited increased IL-17A and IL-17 F compared to singularly infected IL-27Rα−/− mice, while only elevated IL-17 F production was seen between WT and IL-27Rα−/− co-infected mice. IL-27Rα−/− co-infected mice had attenuated IL-10 levels compared to WT controls. Finally, there was a significant difference in bacterial burden between dual infected IL10−/− and WT mice. These data suggest that IL-27 induces IL-10 and suppresses IL-17, which may predispose mice to exacerbation of *S. aureus* pneumonia during influenza infection. IL-10 may also protect against the development of lung injury during co-infection. These observations reveal an important mechanism by which influenza alters host defense and predisposes to secondary infections. Further, IL-27 may play a dual role in co-infection immunopathology; limiting inflammation and tissue injury, while increasing susceptibility to secondary bacterial pneumonia.

IL-27 inhibits multiple T-cell pathways, including Type 17 responses [6]. Type 17 immunity activates neutrophils and antimicrobial peptides via its signature effector cytokines IL-17 and IL-22 in response to stimulation by multiple signals, including IL-23, IL-1β, and IL-6. Prior work has demonstrated that IL-17 is required for acute lung injury during influenza infection [22]. IL-17R−/− mice challenged with a lethal dose of influenza A had decreased weight loss and IL-17RA−/− mice decreased mortality compared to controls. Although lung injury was decreased, IL-17RA−/− mice did not exhibit any failure to clear influenza virus from the lung. In addition, IL-17RA was dispensable for T cell specific recruitment to influenza hemagglutinin or nucleocapsid protein [22]. IL-27 mediated suppression of IL-17 may represent an important pathway limiting influenza induced inflammation. In our current study, IL-27Rα−/− mice challenged with a non-lethal dose of influenza A had increased morbidity compared to controls but no change in viral burden, suggesting that IL-27 is dispensable for viral clearance. IL-27 was necessary to protect against weight loss during influenza infection. These findings are consistent with recently published data by Liu, et. al, which demonstrated accelerated weight loss in IL-27Rα−/− mice during influenza infection [23]. Prior studies have shown that IL-27 potently induces IL-10 production by immune cells. In response to respiratory virus infection, IL-10 is made by several cell types including CD8^+^ T lymphocytes. IL-27 has also been shown to cooperate with IL-2 from CD4^+^ helper T cells to induce IL-10 through a Blimp-1 dependent mechanism during influenza infection [24]. Although IL-27 acts to induce IL-10 during primary virus infection, CD8^+^ T cells have a loss of responsiveness to IL-27 during recall response related to an attenuation of the glycoprotein 130 cytokine receptor, resulting in down-regulation of IL-10 production [25]. Multiple recent studies have suggested that IL-10 plays a critical role in the resolution of influenza infection [11,26,27]. In addition to IL-27, type I interferon affects IL-10 production during infection. Type I interferon has been shown to promote resolution of viral load during influenza through IL-10 production [11]. Exogenous IL-10 improves mortality and decreases lung pathology in both WT and IFNαR−/− mice infected with influenza [11,26]. However, IL-10−/− mice have been found to have increased survival compared to WT mice when challenged with influenza, correlating with increased expression of Type 17 immunity associated cytokines [27]. In our study, changes in either IL-17 or IL-10 production (via IL-27 regulation) may have influenced the weight loss seen in the IL-27Rα−/− mice. In addition to mediating IL-17 and IL-10 responses, it has been reported that IL-27 signaling affects IFNγ production by CD8^+^ cells during primary influenza infection [23,28]. In our current study, we observed increased IFNγ protein levels in the influenza-infected IL-27Rα−/− mice compared to WT, confirming that IL-27 signaling attenuates IFNγ production during influenza infection.

We have previously published that influenza A inhibits bacterial immunity through attenuation of Type 17 responses in the murine lung. Preceding influenza A decreases IL-17A^+^ producing cells, including both CD4^+^ and γδ^+^ cell populations in response to secondary *S. aureus* challenge [15]. IL-27 is known to impair Type 17 immunity by inhibiting the differentiation of naïve T cells into T_H_17 cells through STAT-1 dependent suppression of the T_H_17 specific transcription factor retinoid-related orphan receptor (ROR)-γt [8]. In addition, human TCRγδ^+^ lymphocytes express gp130 and WSX-1 chains of the IL-27R and IL-27 may impact function of γδT cells [29]. Cao et al. has recently shown that IL-27 inhibits IL-17A production in γδT cells activated by *Streptococcus pneumoniae* and that post-influenza pulmonary pneumococcal burden in IL-27R-deficient mice are decreased compared to WT controls at both 24 and 48 hours following bacterial challenge [30]. In our current study, preceding influenza enhanced susceptibility to secondary *S. aureus* pneumonia in both WT and IL-27Rα−/− mice, although the bacterial burden was significantly reduced in the co-infected IL-27Rα−/− mice compared to co-infected WT mice. Importantly, co-infected IL-27Rα−/− mice have increased production of IL-17 F and IL-22 compared to co-infected WT mice, suggesting that IL-27 signaling significantly impacts Type 17 immunity during influenza, *S. aureus* co-infection. Type 17 responses are required for effective bacterial clearance in *S. aureus* pneumonia [15]. Thus, IL-27Rα−/− mice that have increased Type 17 cytokine responses compared to controls, have improved bacterial clearance during co-infection. Notably, preceding influenza enhanced susceptibility to secondary *S. aureus* pneumonia in both WT and IL-27Rα−/− mice at a similar magnitude, confirming that IL-27 plays a role in the exacerbation of *S. aureus*, but is not the sole mechanism by which preceding influenza allows for enhanced susceptibility to secondary bacterial infection.

Previous studies have shown that type I interferon promotes resolution of viral load during influenza infection through IL-10 production and exogenous IL-10 improves mortality and decreases lung pathology in IFNαR null mice infected with influenza [11]. Although IL-10 has been shown to improve outcomes during influenza infection, the results of co-infection studies using post-influenza bacterial models have been mixed. Van der Sluijs and colleagues showed that IL-10 mediates increased susceptibility to secondary infection in a post-influenza pneumococcal model [12] while others have observed no differences between WT and IL-10−/− mice in susceptibility to secondary bacterial infections [31,32]. In our current study, IL-27Rα−/− mice displayed increased lung pathology but decreased bacterial burden of *S. aureus* during co-infection. IL-10−/− mice showed significantly decreased *S. aureus* bacterial burden during co-infection, confirming that IL-10 plays a part in post-influenza secondary bacterial pneumonia. This finding is consistent with IL-27 mediating host defense during influenza infection IL-10 dependent and independent pathways [23]. The increased lung pathology seen in the co-infected IL-27Rα−/− mice may be related to lower IL-10 levels compared to WT controls. Although this conclusion differs from some previously published literature [30-32], these studies utilized a model of post-influenza pneumococcal infection compared to our model of post-influenza staphylococcus infection, which may be the reason for the observed differences. In addition to decreased IL-10 levels, the IL-27Rα−/− mice have an increased IL-17 response, which could also contribute to worsened lung histopathology. It is important to note that IL-27 mediated IL-10 and IL-17 production may be variable throughout the time course of post-influenza *S. aureus* pneumonia and further study is required.

## Conclusions

In conclusion, our study supports that IL-27 signaling regulates enhanced susceptibility to *S. aureus* pneumonia following influenza infection, potentially through both the induction of IL-10 and suppression of IL-17. IL-27 signaling contributes to both host defense and lung injury, as it attenuates lung pathology following viral infection, but enhances susceptibility to secondary bacterial. These findings are significant given the mortality associated with post-influenza bacterial pneumonia and reveal a novel mechanism by which influenza enhances susceptibility to secondary bacterial pneumonia. IL-27 signaling may be important to host defense against other pathogens at the mucosal barrier. The identification of mechanisms by which influenza predisposes to complicating bacterial infections may allow for the identification of therapeutic targets in the future.

## Abbreviations

WT: Wild-type
IL-27Rα−/−: IL-27 receptor α knock-out mice
